# Aerobic Physical Exercise as a Non-medical Intervention for Brain Dysfunction: State of the Art and Beyond

**DOI:** 10.3389/fneur.2022.862078

**Published:** 2022-05-13

**Authors:** Yuxiang Jia, Yu Yao, Limin Zhuo, Xingxing Chen, Cuina Yan, Yonghua Ji, Jie Tao, Yudan Zhu

**Affiliations:** ^1^School of Medicine and School of Life Sciences, Shanghai University, Shanghai, China; ^2^Department of Neurology and Central Laboratory, Putuo Hospital, Shanghai University of Traditional Chinese Medicine, Shanghai, China

**Keywords:** aerobic physical exercise, cognition, depression, chronic pain, neuroinflammation, synaptic plasticity, hippocampal atrophy, bone-brain axis

## Abstract

Brain disorders, including stroke, Alzheimer's disease, depression, and chronic pain, are difficult to effectively treat. These major brain disorders have high incidence and mortality rates in the general population, and seriously affect not only the patient's quality of life, but also increases the burden of social medical care. Aerobic physical exercise is considered an effective adjuvant therapy for preventing and treating major brain disorders. Although the underlying regulatory mechanisms are still unknown, systemic processes may be involved. Here, this review aimed to reveal that aerobic physical exercise improved depression and several brain functions, including cognitive functions, and provided chronic pain relief. We concluded that aerobic physical exercise helps to maintain the regulatory mechanisms of brain homeostasis through anti-inflammatory mechanisms and enhanced synaptic plasticity and inhibition of hippocampal atrophy and neuronal apoptosis. In addition, we also discussed the cross-system mechanisms of aerobic exercise in regulating imbalances in brain function, such as the “bone-brain axis.” Furthermore, our findings provide a scientific basis for the clinical application of aerobic physical exercise in the fight against brain disorders.

## Introduction

Physical exercise (PE) is a non-medical intervention that has been strongly validated by systematic reviews, statistical analyses, clinical examinations, and appropriate guidelines (1–3). Appropriate PE contributes to numerous physiological and psychological benefits, as well as a reduced tendency to develop chronic diseases, such as cardiovascular, cerebrovascular, and metabolic diseases (4, 5). PE can be divided into aerobic and resistance PE. The former includes running or cycling (6), which is better for cardiopulmonary health and reduces hippocampus decline (7–9). The latter improves bones and muscles by resisting external resistance through increased muscle strength (10). Therefore, in some chronic diseases, such as chronic heart failure (11), multiple sclerosis (12), and depression in older adults (13), PE may be a first-line treatment option.

The aerobic PE emphasized in this paper can be classified according to maximal oxygen uptake (VO_2max_) as low- (<45% VO_2max_), moderate- (45–64% VO_2max_), and high-intensity (70–85% VO_2max_) PE (14). The American College of Sports Medicine stated that moderate-intensity aerobic PE performed five or more days per week with complementary resistance exercises performed 2 or 3 days per week is beneficial to human health (15, 16). In addition, aerobic PE has two training methods: voluntary PE and forced PE. The former is a long-term self-development and self-sustainment therapy (17). The experimenters created voluntary animal models in an environment equipped with a running wheel (18), maze, or climbing gear (17). The latter refers to the voluntary use of mechanical assistance to achieve and maintain an ideal state of motion (19). Treadmills (20) and forced wheel-running (21) are often used in animal experiments to simulate this mode of movement. Forced PE can precisely control exercise intensity, and may be a better method to study the effects caused by different PE intensities, although cannot avoid individual differences among mice (22). A voluntary PE environment encourages mice to engage in low-intensity PE, such as free running; however, researchers are unable to control the amount of exercise performed (22). Moreover, forced PE seemed to produce more bromodeoxyuridine+ cells, although it increased anxiety-like behaviors in the animals (23). For some patients who are unable to perform voluntary PE, researchers first chose mechanically-assisted forced PE (19, 24–26).

Increasing evidence in recent years has focused on the notion that PE has positive effects on cognitive impairment, depression, and chronic pain. The first chapter of this paper confirms this fact using examples of some common neurodegenerative diseases that are associated with cognitive impairment, such as Alzheimer's disease (AD) (27) and Parkinson's Disease (PD) (28). In the case of AD, we have presented several studies that provide preclinical/clinical evidence (29, 30) supporting the recovery effect of aerobic PE on cognitive impairment. Next, we focused on depression. Depression is a common mood disorder that affects ~300 million people worldwide and was likely worsened in recent years because of the coronavirus disease 2019 pandemic, which exacerbated chronic stress related to work and school (31–33). Currently, the approach to managing depression is singular, and antidepressant drugs are typically used; however, this kind of drug treatment is ineffective for some patients who have a poor response and are likely to experience pharmacological side effects (34). The beneficial effects of aerobic PE on depression are clear (2, 35). Finally, we summarized the intervention effects of aerobic PE on chronic pain. Chronic pain is a serious threat to the health of the elderly, and has serious adverse effects on their physical, psychological, and social functions and increases the incidence of other complications in this population (36). There is an abundance of evidence that aerobic PE is a viable treatment option for chronic pain (37, 38), not only to improve the pain symptoms, but also to alleviate comorbidities such as sleep disturbances and poor memory (39, 40).

Chronic brain diseases may potentially share underlying pathophysiological mechanisms. In the second part of this paper, we discuss neuroinflammation (41–43), synaptic plasticity (43, 44), hippocampal volume, and neuronal apoptosis, which are associated with the pathological occurrence and development of common brain diseases, and clarify their relationships with aerobic PE. A genome-wide association study showed a high degree of genetic overlap between several mental disorders and pointed out that different mental disorders are not separate diseases but different overlapping phenotypes of the same clinical spectrum (35). Therefore, the use of the appropriate exercise types and intensities to intervene in a variety of brain diseases provides certain theory basis.

The brain has always been regarded as the “commander” of various organs, whereas bones have always been regarded as the “protector” and “supporter” of the human body. There seems to be no relationship, although recent studies have shown bilateral functional dependence between the two (45, 46). It is well-established that the brain influences bone regeneration and homeostasis through “efferent nerves” (47–49), and evidence is increasing that bones interfere with brain homeostasis through “afferent nerves” (50, 51). Bones are the main operators of exercise, and the beneficial effects of aerobic PE on bones have been proven (52, 53). Therefore, in the third part of this paper, we discuss several bone-derived proteins that may change brain function, and link them to Piezo1, a popular mechanical ion channel. Based on the above theories, this paper proposes a hypothesis that aerobic PE interferes with brain diseases through the bone-brain axis.

## Aerobic PE Improves Various Functional Modalities

### Aerobic PE Improves Cognitive Function

It is well-known that cognitive function declines with age, and the positive effects of aerobic PE on this decline have been well-demonstrated in rodents (54). In animal models of neurodegenerative diseases, including AD (55) and PD (56), PE has been repeatedly shown to up-regulate adult hippocampal neurogenesis and promote cognitive improvement in the aging brain (57). Among humans, the powerful benefits of aerobic PE are reflected decisively and vividly in the elderly (58). Compared with the sedentary elderly population, older adults who engage in PE have shown significant differences in bone mineral density, muscle content, and especially cognitive function (59). For instance, magnetic resonance imaging showed increased gray and white matter volume in the anterior cingulate cortex after 6 months of aerobic PE (60 min, 3 days per week) (60). In addition, aerobic PE is also beneficial in preventing AD. Older adults who were sedentary had a 53% higher prevalence than older adults who were more active (hazard ratio = 0.477, 95% confidence interval: 0.273–0.832) (61). The large, single-blinded, multi-center study showed that 16 weeks of aerobic PE increased oxygen volume (a marker of cardiorespiratory fitness) by 13%, leading to improvements in cognitive and neuropsychiatric symptoms (62). Similarly, aerobic PE reduces the progression of PD. Studies have shown that 6 months of aerobic PE leads to increased functional connectivity of the anterior putamen with the sensorimotor cortex relative to the posterior putamen and enhanced cognitive performance (63).

From the examples above, aerobic PE requires long-term adherence to show an advantage in terms of improving cognitive function. However, there is evidence that aerobic PE interventions do not improve symptoms in all age groups, such as the 60–80-year-old population (64–66). These contradictory results can be explained by the varying optimal types and intensities of PE among different age groups (67). Therefore, aerobic PE provides a low-cost and widely available intervention for improving cognition in the elderly, especially patients with AD.

### Aerobic PE Can Fight Depression

Depression is a common mental disorder that threatens the physical and mental health of people worldwide and is a major cause of rising suicide rates in the 21st century (68). Doctors mainly diagnose the symptoms of some patients according to the Diagnostic and Statistical Manual of Mental Disorders (69) and International Classification of Diseases (70). However, there is little evidence on the mechanism of aerobic PE in regulating depression. Previous studies have shown that stress increases levels of kynurenic acid in the plasma and brains of mice (71) and leads to inhibited serotonin synthesis; mice could reduce the inevitable sense of helplessness caused by stress by reducing plasma kynurenic acid through 4 weeks of wheel running (71). Compared with the trained mice, mice without wheel running showed a stronger sense of helplessness in the tail suspension, escape, sugar water preference, and forced swimming tests (72). Notably, a similar phenomenon has been observed in human studies. Trivedi et al., based on clinical studies, suggested that 12 weeks of high-intensity exercise (70–85% maximum heart rate) was beneficial in reducing depression levels according to the Hamilton Depression Scale (*P* < 0.001) (73, 74).

Current research proves that aerobic PE increases the proportion of gray matter volume in the brain, improves the spatial structure of white matter, and leads to greater functional connectivity in the brain regions associated with major depression (75); its therapeutic effect was similar to that of antidepressants (76). The World Health Organization and National Institute for Health and Care Excellence guidelines recommend that for patients with mild to moderate depression, moderate aerobic PE should be performed in addition to standard drug treatment.

### Aerobic PE in the Treatment of Chronic Pain

Recent statistics show that chronic pain affects 1.5 billion people worldwide, and that these numbers are steadily rising (77). Chronic pain is often accompanied by spontaneously progressive symptoms, including depression, anxiety, sleep disorders, intellectual disability, and anorexia, resulting in decreased quality of life for patients (78, 79). The 2021 International Association for the Study of Pain meta-analysis of 460 patients used a quality effects model. The Physiotherapy Evidence Database (PEDro) scale showed that the combination of pain neuroscience education and 12 weeks exercise had greater short-term improvements in chronic musculoskeletal pain severity, disability, kinesiophobia, and pain catastrophizing compared with those of exercise alone (80).

The core mechanism of aerobic PE in chronic pain is to inhibit local and systemic inflammation and prevent central sensitization (81–83). On the one hand, prolonged sitting leads to an imbalance in the proportion of cytokines in local and systemic circulation, resulting in a hyperinflammatory state that contributes to the onset and maintenance of chronic pain (84). Aerobic PE can reduce systemic inflammation and presence of proinflammatory cytokines and up regulate anti-inflammatory cytokines, allowing the neuroimmune signals in the central nervous system to be normalized. Chronic pain can be reduced, or hyperalgesia can be prevented and reversed (85, 86). On the other hand, in healthy people, aerobic PE releases endogenous opioids and acts as a pain reliever, an effect called “exercise-induced analgesia” (86, 87). Unfortunately, this mechanism does not apply to all patients with chronic diseases, as exercise-induced analgesia may be insensitive or even missing in some chronic pain diseases, such as fibromyalgia and chronic fatigue syndrome (88, 89). Furthermore, chronic pain is caused by genetics (90), stress, or sedentary (91) imbalances in central neurotransmitters such as serotonin, dopamine, and norepinephrine, whereas PE triggers a stress response in the neuroendocrine system, thereby changing the balance of these neurotransmitters (92).

## Aerobic PE Maintains Brain Homeostasis Through Regulatory Mechanisms

Some studies have shown that exercise can reduce symptoms in people with brain damage (93–95). Unfortunately, while the benefits of exercise on brain and cognitive function are well-known, the mechanisms behind it not always been clear. Various chronic brain diseases have the same potential mechanisms. Here, we summarized several mechanisms of aerobic PE in alleviating brain diseases, including anti-inflammatory mechanisms, synaptic plasticity, hippocampal volume, and the apoptosis pathway of hippocampal neurons.

### Effect of Aerobic PE on Brain Inflammation Through Anti-inflammatory Mechanisms

Microglia is the monitor and regulator of neuroinflammation (96). When the body endures a pathological injury, the microglia can be activated by pro-inflammatory factors (97) to mediate downstream signaling pathways that trigger inflammatory reactions and aggravate inflammation (98), or inhibited by anti-inflammatory factors that restore the body to homeostasis (99–101). Exercise can regulate microglial activity and inhibit neuroinflammation by increasing anti-inflammatory factors (102, 103). There are numerous examples that support this idea, such as animal studies that tested interleukin-6 (IL-6) (103, 104), interleukin-10 (IL-10) (105, 106), and CD200-CD200R (107, 108) levels before and after exercise; clinical trial data suggest the same thing (109, 110).

In addition to the anti-inflammatory factors mentioned above, Prof. Tony Wyss-Coray and his team found that the blood of mice produced the protein clusterin after one month of running on the wheel, which inhibited brain inflammation and promoted a large increase in the number of neurons and other cells, thereby improving cognitive impairment (111).

### Effects of Aerobic PE on Synaptic Plasticity Through Neurotrophic Factors

Synaptic plasticity refers to the activity-dependent change in neuronal connection strength (112). Long-term potentiation is a persistent, activity-dependent increase in synaptic strength that occurs in response to repeated synaptic stimuli and is considered a common cellular manifestation of learning and memory. Some studies have confirmed that rats and mice undergoing running programs showed increased long-term potentiation at synapses in the hippocampus (68, 113), however, this has rarely been reported in clinical trials.

Aerobic exercise enhances synaptic plasticity in a variety of ways (114–116). For example, increased neurotrophic factors such as brain-derived neurotrophic factor (BDNF) and insulin-like growth factor 1 (IGF-1) induced by exercise training play an important role in promoting synaptic plasticity. BDNF can induce neurogenesis in the dentate gyrus of the hippocampus (117) and increase synaptic plasticity through calcium and calmodulin dependent kinase-mitogen-activated protein kinase activation mediated by tropomyosin receptor kinase B and N-methyl-D-aspartate receptors, followed by cAMP response element-binding protein activation (117). However, when tropomyosin receptor kinase B of BDNF was blocked during exercise, cognitive performance was impaired, and synaptic proteins in the hippocampus were reduced (118). In sedentary rats, upregulation of BDNF had a positive effect on the hippocampus (118). Therefore, BDNF plays an important role in synaptic plasticity (119). Similarly, intra hippocampal injection of IGF-1 functional blockers in mice demonstrated that IGF-1 signaling plays an important role in the effect of exercise on hippocampal dependent learning and plasticity (120).

### Aerobic PE Intervenes in Brain Diseases by Preventing Hippocampal Atrophy

Hippocampal volume is a vital indicator of brain health that decreases with aging and neurological diseases, such as severe depression (121) and schizophrenia (122). The mechanisms involve a variety of molecular or cellular structures (123). Animal and clinical studies have consistently demonstrated that PE can effectively alleviate hippocampal atrophy (124–126). Moreover, a detailed study showed that in people with cognitive and mental health disorders (including younger and older people), proper aerobic PE for more than 6 months had a positive effect on hippocampal volume even in elderly people who are vulnerable to hippocampal atrophy (124). Participants returned to baseline levels after 6 weeks of inactivity, indicating that long-term aerobic PE is important for maintaining exercise-induced changes in hippocampal volume (127). In addition, although BDNF expression induced by aerobic PE is positively correlated with changes in hippocampal volume, there is no convincing evidence of the relationship between them (128, 129).

### Aerobic PE Intervenes in Brain Diseases by Inhibiting Hippocampal Neuronal Apoptosis

Aerobic PE has two effects on hippocampal neuronal apoptosis. First, moderate exercise slows down hippocampal neuron injury caused by stress and inhibits neuronal apoptosis. Some studies have shown that the exocrine body derived from circulating endothelial progenitor cells can protect endothelial cells from hypoxia, and that moderate aerobic PE can enhance its function (130). C57BL/6 mice received moderate treadmill exercise (10 m/min) for 4 weeks following middle cerebral artery occlusion stroke. Compared with the control group, the apoptosis rate of trained mice decreased by 40% (131). In a D-galactose-induced aging rat model, swimming reportedly reduced Fas- and mitochondrial-dependent apoptotic pathways, significantly inhibited inflammatory signal activity, and also enhanced hippocampal survival pathways. Therefore, swimming can reduce the brain apoptosis and inflammatory signal activity induced by aging (132).

Excessive exercise can lead to cell damage and pathological apoptosis in multiple tissues and organs of the body. Besides skeletal muscle and cardiomyocytes, excessive exercise-induced injury and apoptosis were also found in hepatocytes, renal tubular cells, and lymphocytes in non-exercise systems (133). Currently, the research on nerve cell injury and apoptosis caused by excessive exercise is still in its infancy, mainly in the hippocampus. It can be observed that excessive exercise, like hunger, trauma, and other stressors, is a type of stress for the human body. Although moderate exercise causes benign stress that is beneficial to the human body, excessive exercise can lead to excessive stress, which causes the arrangement of hippocampal neurons to become loose and disordered with reduced communication and nuclear pyknosis, resulting in multi-system damage (134, 135) that can even lead to overtraining syndrome (136).

## The Molecular Mechanism of Aerobic PE Regulating the Brain Through the Bone-Brain Axis

Bone and skeletal muscle are the two major organs mainly affected by exercise in the body; bones are regarded as scaffolds that support and protect various organs in the body (137). Muscles transmit and receive the mechanical force caused by exercise and are therefore also considered an endocrine organ (138). Piezo1 in the Piezo family is a mechanically activated ion channel (139) that acts as a mechanical sensor in osteoblasts and osteocytes. It is beneficial to the formation of bone trabeculae in the process of endochondral ossification and reportedly increased the bone thickness of mice (140, 141) and mediated the Piezo1/Yes-associated protein1 (YAP1)-collagen pathway to indirectly regulate the bone resorption activity of osteoclasts, thus affecting bone metabolism (141). Moreover, mechanical unloading can inhibit the expression of piezo and slow down osteoblast and bone formation. A previous study found that Piezo1 promoted the expression of Wnt1 in osteocytes by activating YAP1 and transcriptional coactivator with PDZ-binding motif (TAZ) (142). Piezo1 activates the Wnt1 signal pathway in osteocytes and leads to increased bone formation and decreased bone resorption (142). Recently, Sasaki et al. found that Piezo1, a mechanically-sensitive ion channel, mediated the phosphorylation of protein kinase B in osteocytes and down-regulated the expression of sclerosing proteins (143). Because sclerostin leads to bone mass loss, Piezo1 in osteocytes inhibits the expression of sclerosis proteins and promotes osteoblast formation by activating the protein kinase B signal pathway. Bone tissue regulates brain function mainly through osteoblast secretion of a variety of proteins (Table 1), including osteocalcin (OCN), lipid delivery protein-2 (LCN2), and osteopontin (OPN); cells such as bone-derived mesenchymal stem cells, hematopoietic stem cells, and microglia-like cells are also provided (144).

**Table 1:** Bone-derived proteins involved in brain disorders.

**Proteins**	**Function**
OCN	Regulates insulin secretion and testosterone production; promotes muscle adaptation to exercise; increases the release of serotonin, dopamine, and norepinephrine; and inhibits the release of γ-aminobutyric acid, thereby reducing depression and anxiety-like behaviors.
LCN2	Increases neuroinflammation, decreases amyloid-β plaque clearance, and decreases dehydrogenase activity and survival rate of wild-type astrocytes.
OPN	Reduces amyloid-β plaque and malnourished neurites; increases angiogenesis and differentiation into functional dopaminergic neurons; and decreases microglial activation and loss of tyrosine hydroxylase positive neurons.

*OCN, osteocalcin; LCN2, lipid delivery protein-2; OPN, osteopontin*.

### OCN

OCN, also known as bone γ-carboxyl glutamate protein, is uniquely secreted by osteoblasts (145). It plays an important role in the regulation of bone calcium metabolism and is a new biochemical marker in the study of bone metabolism, which has important value in the diagnosis of osteoporosis syndrome (146) and other diseases such as abnormal calcium metabolism (144, 147). In the peripheral nervous system, OCN binds to G protein-coupled receptor family C group 6 (Gprc6a), and regulates hormone levels, including insulin and testosterone, to promote skeletal muscle adaptation to PE. In the brain, OCN binds to G protein-coupled receptor 158 (Gpr158) and can cross the blood-brain barrier (BBB) to regulate transcription factors in the ventral tegmental area (VTA), dorsal raphe nucleus, middle raphe nucleus, and hippocampal CA3 neurons, thereby increasing the release of serotonin, dopamine, and norepinephrine, inhibiting the release of γ-aminobutyric acid (148, 149), and reducing depression and anxiety-like behaviors. Gpr158 was the first OCN receptor found in the brain. It is present in the somatosensory, motor, and auditory areas of the cortex, as well as in the piriform cortex, hippocampus, post splenic area, and ventral tegmental area. Significant decreases in OCN levels in elderly mice with cognitive impairment and patients with depression have been reported (150), although injection of exogenous OCN can reverse these defects (148).

### LCN2

LCN2 is another hormone known as a neutrophil gelatinase-associated lipid carrier protein, which is a secretory glycoprotein (151). LCN2 was previously considered to be a lipid-derived factor (152), however the expression profile showed that the expression of LCN2 in bone was 10 times higher than that in adipose or other tissues; therefore, it is also a bone-derived factor (50). Similar to OCN, LCN2 acts directly on β cells to promote their proliferation as well as insulin secretion (153). Recently, researchers at Columbia University Medical Center found that LCN2 proteins secreted by osteocytes not only induce insulin secretion, but also cross the BBB and activate the anorexigenic (appetite-suppressing) pathway by binding to melanocortin 4 receptors in the hypothalamus, thereby controlling body weight, fat content, and insulin sensitivity (50). LCN2 also enhances neuronal motor and inflammatory responses by activating Janus kinase 2-activator of transcription-3 crosstalk (154) and nuclear factor kappa B pathways (155) to up-regulate the expression of chemokine (C-X-C motif) ligand 10 (156). LCN2 levels in brain tissue and astrocyte cultures of rats with ischemic stroke and astrocytes treated with standardized hypoxia were reportedly significantly increased, while BBB permeability, neurological impairment, cerebral infarction, and neutrophil infiltration were decreased in LCN2-deficient rats (157, 158). Further studies found that LCN2 promotes neuroinflammation by activating neutrophil infiltration, microglia, and astrocytes and inducing proinflammatory cytokines and chemokines (159–161). These results suggest that LCN2, neuropathology, and PE are inextricably linked.

### OPN

OPN is a secretory stromal cell protein found in bones (162). It was subsequently proven to be expressed in epithelial lining, skeletal muscle, and breast and brain tissue (163, 164). OPN plays an important role in tissue remodeling, immunomodulation, and biomineralization by binding to diverse receptors, such as integrins and CD44 (165–167). In bone tissue, OPN can anchor osteoclasts to the bone mineral matrix to promote bone resorption (167–169); therefore, patients with high serum OPN concentrations have low bone mineral densities, whereas patients with low serum OPN concentrations have high bone mineral densities (170). In the brain, OPN forms different fragments after protease cleavage. These fragments can bind to different receptors (CD44 and integrin) to activate P42/44 mitogen-activated protein kinase and phosphoinositide 3 kinase pathways that have a neuroprotective function (171). OPN can also activate c-Jun N-terminal kinase and extracellular regulated protein kinase pathways to cause neuroinflammation by up-regulating proinflammatory gene expression (172, 173). In addition, OPN can act as a pro-inflammatory cytokine to recruit inflammatory cells to the lesion site and cause nervous system disease (174, 175). Therefore, OPN is an important factor in regulating bone mass and triggering neuroinflammation, and it is particularly crucial to explore the secretion mechanisms of OPN regulated by aerobic PE.

## Conclusion

**Figure 1:**
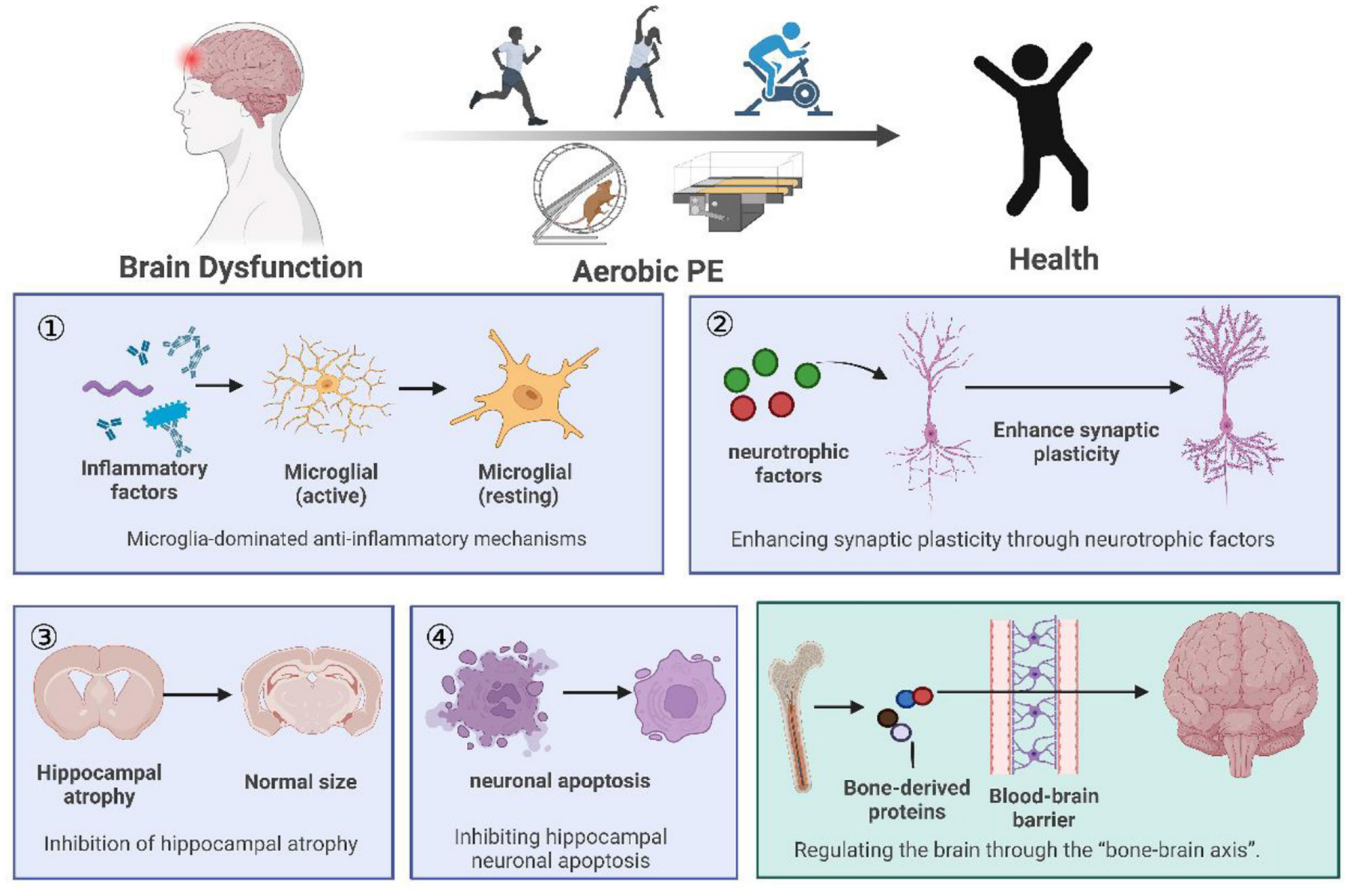
Summary of the review. (1) We listed animal and clinical experiments on the intervention of aerobic PE in brain dysfunction, and concluded that aerobic PE can improve cognition, fight depression, and relieve chronic pain. (2) Aerobic PE interferes with brain disorder through four common mechanisms: anti-inflammatory mechanisms, synaptic plasticity, hippocampal atrophy, and hippocampal neuronal apoptosis. (3) We propose the hypothesis that aerobic PE interferes with brain disease through the bone-brain axis.

**Figure 2:**
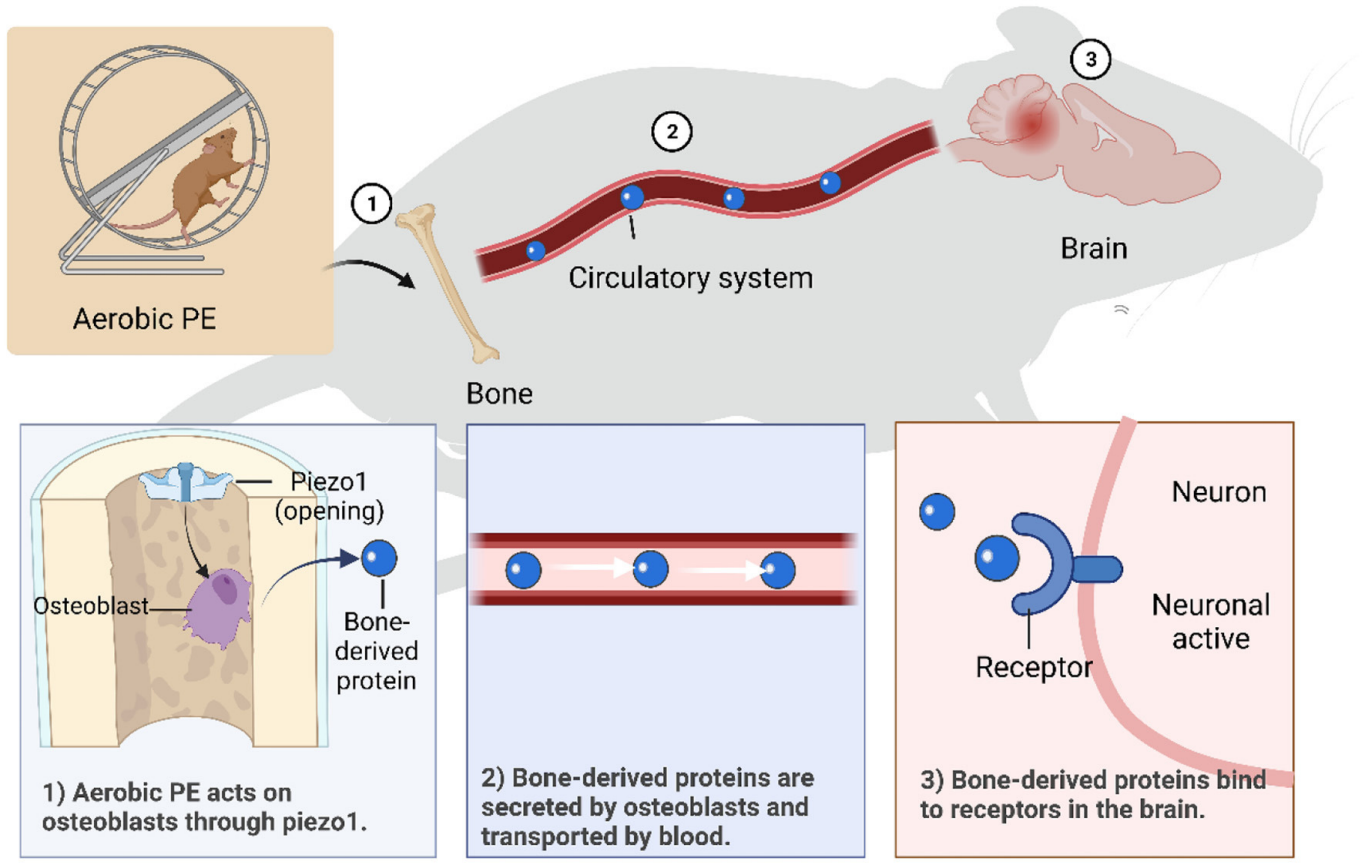
Mechanisms of aerobic PE intervening in brain dysfunction through bone-brain axis. (1) Piezo1 is activated by aerobic PE, which promotes osteoblast growth and secretion of bone- derived proteins. (2) Bone-derived proteins are transported in blood and can cross the blood-brain barrier. (3) Bone-derived proteins bind to specific receptors on neurons to function.

Human and animal studies have shown that aerobic PE has significant effects on many aspects of brain function, including preventing and improving cognitive function, depression, and chronic pain. In this paper (Figure 1), we discussed four regulatory mechanisms of aerobic PE in the intervention of neurological diseases, including anti-inflammatory pathways related to microglia, promotion of hippocampal synaptic plasticity through a variety of neurotrophic factors, and prevention of hippocampal atrophy and neuronal apoptosis. Furthermore, we have introduced a novel mechanism of bone-brain axis regulation (Figure 2), although its research is still in its infancy.

An appropriate amount of aerobic PE activates beneficial mechanisms in the body. Effective aerobic PE causes tissues to release IL-1α to activate ion channel Piezo1 (176) and promotes osteoblast formation through integrin β1 and integrin-focal adhesion kinase pathways (177). As previously mentioned, bone tissue regulates inflammation mainly by secreting a variety of factors related to neuroinflammation, including OCN (147) and OPN (170), and promotes subdivided secretion of cytokines, such as bone-derived mesenchymal stem, hematopoietic stem, and microglia-like cells (178). Therefore, regulatory factors from bone can pass through the BBB and regulate transcription factors in neurons in various regions of the brain, thereby increasing the release of related hormones and reducing the occurrence of neuroinflammation. We can conclude that aerobic PE activates Piezo1 through skeletal muscle pressure, promotes osteoblasts to secrete bone-derived proteins, and interferes with related nerve inflammation through the bone-brain axis. However, exercise intensity, time, and frequency are critical to the effects of exercise; painful exercise can aggravate nerve inflammation. Furthermore, acute high-intensity exercise with higher than normal duration or without physical adaptation level induces oxidative stress (179) and muscle injury (180).

In conclusion, it is necessary to establish different exercise intensities, times, frequencies, and even exercise methods for different backgrounds on an individual basis. Although the study carried a large workload, it can have a profound impact as a non-medical intervention.

## Author Contributions

YJia, YY, LZ, XC, CY, YJi, and JT drafted the manuscript and revised it critically for intellectual content. YZ drew the table. All authors read and approved the final version of the manuscript before submission.

## Funding

This work was supported by grants from National Key Research and Development Program (No. 2020YFA0803800), National Natural Science Foundation of China (Nos. 31771191, 82074162, and 81903995), Young Elite Scientists Sponsorship Program by CACM (No. CACM-2019-QNRC2-C10), Project for Capacity Promotion of Putuo District Clinical Special Disease (No. 2019tszb02), Science, Technology Innovation Project of Putuo District Health System (Nos. ptkwws201902, ptkwws201908, and ptkwws202107), and the One Hundred Talents Project of Putuo Hospital, Shanghai University of Traditional Chinese Medicine (No. 2022LH002).

## Conflict of Interest

The authors declare that the research was conducted in the absence of any commercial or financial relationships that could be construed as a potential conflict of interest.

## Publisher's Note

All claims expressed in this article are solely those of the authors and do not necessarily represent those of their affiliated organizations, or those of the publisher, the editors and the reviewers. Any product that may be evaluated in this article, or claim that may be made by its manufacturer, is not guaranteed or endorsed by the publisher.
